# Assessment of Fixed Bearing and Rotating Platform Total Knee Arthroplasty Designs Using the Patient Knee Implant Performance Scoring System: Post-hoc Pooled Analysis of Data From Two Prospective, International, Multi-centre, Non-randomised Studies

**DOI:** 10.7759/cureus.109613

**Published:** 2026-05-25

**Authors:** Naseer Mohammed Abdul, Thierry Bernard, Piers Yates

**Affiliations:** 1 Orthopaedics, Fiona Stanley Hospital, Perth, AUS; 2 Orthopaedics, Fremantle Hospital, Perth, AUS; 3 Biostatistics, DePuy Synthes, West Chester, USA

**Keywords:** bmi, fixed bearing, proms, rotating platform, total knee arthroplasty

## Abstract

Background

The optimal choice of polyethylene insert in total knee arthroplasty (TKA) continues to cause contention. Rotating platform bearings (RP) have theoretical advantages but have failed to demonstrate a functional difference compared to fixed bearings (FB) in clinical studies. No studies have analysed the effect of body mass index (BMI) on functional scores between RP and FB TKA. No studies have investigated RP and FB TKA using the new Patient Knee Implant Performance (PKIP) patient-reported outcome measure (PROM).

Methods

This was a post-hoc analysis of the data from two prospective, non-randomised, multi-centre studies conducted across centres in Australia, the United Kingdom, and the United States by DePuy Synthes (West Chester, Pennsylvania, United States). Functional outcomes between RP TKA and FB TKA were compared. Baseline and two-year follow-up data were compared across multiple PROMs in both groups, including PKIP. The analysis included adjustment for BMI between groups.

Results

A total of 1,718 patients across 27 centres were included in the study. Of this, 959 participants underwent FB TKA and 759 participants underwent RP TKA. No significant difference was found between RP and FB in the change from baseline at two years across any parameter in all PROMs. When adjusted for BMI, statistical differences were seen between RP TKA and FB TKA at two years in several PROMs.

Conclusion

Both FB and RB showed excellent results at two years. Our data does not demonstrate a significant difference in RP TKA over FB TKA at two years in any PROM. Advantages were seen in some PROMs for RP when adjusted for BMI alone at two years.

## Introduction

Total knee arthroplasty (TKA) is a reliable and reproducible form of treatment for patients with end-stage osteoarthritis, with over 78,000 TKAs performed in Australia in the year 2024 alone [1]. Despite improved survivorship rates, aseptic loosening remains one of the major causes of revision of TKA [2]. In addition, there remains a high proportion of patients dissatisfied with their TKA, with reports ranging from 12.7% to 20%, despite ongoing innovation in implant design [3]. Whilst multiple factors may contribute to this, a component of the TKA that has continued to be debated is the design of the polyethylene insert.

Rotating platform bearings (RP) have a theoretical advantage when compared to fixed bearings (FB) by reducing contact stress between surfaces, leading to reduced rates of wear and loosening [4]. In RP, the insert can rotate around an axis and theoretically mimic the physiological kinematics of the knee, thus reducing force through the tibial prosthesis [5]. In vivo biomechanical studies have demonstrated an advantage in RP systems compared to FB systems with regard to wear rates [6]. RP is part of a larger group of ‘mobile bearings’, but the RP controls motion on both the upper and lower polyethylene interfaces.

Despite this theoretical advantage, no clinical studies thus far have demonstrated a difference in terms of functional or radiological outcome between RP TKA and FB TKA [7]. All randomised studies have been limited by their small sample size [8,9] and have utilised traditional patient-reported outcome measures (PROMs). Additionally, whilst the index theoretical advantage of RP TKA is reduced wear, no study has investigated the differences between RP TKA and FB TKA when adjusted for body mass index (BMI). A recent large registry-based analysis has, however, suggested higher rates of revision and mechanical loosening associated with MB designs when compared to FB TKA, particularly in longer-term follow-up from national registries [10]. This registry finding contrasts with smaller clinical studies focused on functional outcomes and highlights the need to better understand whether differences exist in patient-reported performance, particularly in specific patient subgroups.

PROMs are questionnaires completed by patients to assess the degree of difficulty or symptom severity. With regard to TKA, whilst some PROMs are generic overviews of a person’s health, such as the EuroQol five-dimensional questionnaire (EQ-5D) [11], specific PROMs exist for the assessment of knee joints. Commonly used PROMs in TKA studies include the Oxford Knee Score (OKS) [12], the Knee Injury Osteoarthritic Outcome Score (KOOS) [13], and the American Knee Society (AKS) score [14].

The conventional knee scoring systems have limitations in that they do not reflect outcomes or measure aspects important to patients, such as confidence whilst performing activities, the perceived stability of the knee, and overall satisfaction with their joint replacement. They also often have a ceiling effect, which reduces the ability to differentiate between better levels of knee performance, and are subject to observer bias [15,16]. New scoring systems have been developed to address these limitations, such as the Patient Implant Knee Performance (PKIP) [17]; however, this instrument has not yet been utilised in comparing RP TKA and FB TKA.

The primary objective of this study was to compare two-year patient-reported functional outcomes between RP TKA and FB TKA using the PKIP PROM. Secondary objectives were to compare functional outcomes across additional validated PROMs and to explore whether BMI influenced functional outcomes between implant designs

## Materials and methods

This study was a post-hoc comparative observational analysis of data derived from two prospective, non-randomised, multi-centre clinical studies [18,19], which were run and conducted by DePuy Synthes (West Chester, Pennsylvania, United States) as part of routine product evaluation. Participants were recruited from across sites in the United States, the United Kingdom, and Australia from November 14, 2012, to May 27, 2015. Participants were followed for two years. Approval was obtained from each participating centre’s Institutional Review Board or ethics committee, and informed written consent was obtained from all participants before enrolment into the studies. The current study was approved by the Western Institutional Review Board (WIRB PRO NUM 20121213/20111599).

Participants were recruited if they were undergoing TKA with either the P.F.C. SIGMA™ Knee System (DePuy Synthes) or the original ATTUNE™ Primary Knee System (DePuy Synthes). Surgeons participating in this study were experienced with these implants and used them in their daily practice. Baseline demographics, preoperative interventions, and PROMs were collected within 90 days of surgery. All participants had standard postoperative care as per their institution and were followed up for two years. PROMs collected were: KOOS [13], OKS [12], EQ5D-3L [20], Knee Society Score (KSS) [21], and PKIP [22]. 

The PKIP Questionnaire was developed to address limitations of traditional knee PROMs. PKIP scores the performance of the knee prosthesis across five subscales [17]. These subscales assess the patient’s awareness of the implant, their confidence with the knee, their sense of stability during activities, the modification of any activities, and satisfaction with knee functions. It comprises 24 items which employ a 5-, 6-, or 11-point ordinal response scale, where higher scores indicate better knee function, and an overall score ranging from zero to 100. PKIP is a highly responsive PROM that has undergone psychometric validation and has demonstrated the capability of discriminating between groups with better or worse knee function [22].

All PROMs used in this study are validated instruments. The KOOS was available free at the time of our study (it was free till 2023), a license agreement was done with Oxford University Innovation Limited for the use of the OKS, and the KSS was also used with permission from the Knee Society. Use of the EQ-5D-3L was conducted in accordance with licensing requirements from the EuroQol Research Foundation. 

Data analysis

All data were analysed using SAS software version 9.4 (SAS Institute Inc., Cary, North Carolina, United States). Demographic characteristics were compared using the two-sided independent t-test for continuous variables and using Fisher’s exact test for categorical variables. Survival estimates and confidence intervals were produced using the Kaplan-Meier methodology, and p-values comparing FB to RP were produced using the log-rank test to analyze rates of revision of any component and rates of revision with removal of either the tibial and/or the femoral component.

The change from baseline (CFB) was calculated as follow-up minus the preoperative measurement. Adjusted changes from baseline were adjusted by using a propensity score methodology. Age, sex, BMI, country, product (PFC SIGMA/ATTUNE), and three baseline ligament indicators (previous treatments: Arthroscopy/Lateral Release/Ligament Reconstruction) were used to establish propensity scores, which predict the subjects’ design type (FB or RP). 

For each PROM separately, propensity scores as well as baseline PROM and design type were then used as covariates in a general linear model to estimate the adjusted change from baseline at two years. BMI was considered a potential confounder influencing functional outcomes following TKA. Therefore, BMI was incorporated into propensity score adjustment models, and a secondary analysis examining BMI-adjusted outcomes was also performed. Propensity scores were generated using logistic regression with implant design as the dependent variable and clinically relevant baseline covariates included in the model. Missing data were handled using complete-case analysis. Centre- or surgeon-level clustering adjustments were not performed.

## Results

A total of 1,718 participants from 27 centres across Australia, the United States, and the United Kingdom were included in this analysis (Table 1). Of these, 959 participants underwent FB TKA and 759 participants underwent RP TKA; 85% of participants in FB TKA and 81% of participants in RP TKA completed two-year data sets. 

**Table 1:** Demographics and breakdown FB: fixed bearings; TKA: total knee arthroplasty; RP: rotating platform bearings

Characteristics	FB TKA (n=959)	RP (n=759)	
Gender, n (%)			
Female	564 (58.8%)	442 (58.2%)	
Male	395 (41.2%)	317 (41.8%)	
Age			
n	959	759	
Mean (SD)	66.1 (7.8)	64.3 (8.1)	
Range	39, 80	28, 85	
BMI			
n	954	759	
Mean (SD)	31.9 (5.9)	32.3 (6.2)	
Range	16.7, 55.4	16.5, 56.1	
BMI >30			
Yes	459 (47.9%)	304 (40.1%)	
No	500 (52.1%)	455 (59.9%)	
BMI >40			
Yes	94 (9.9%)	69 (9.1%)	
No	860 (90.1%)	690 (90.9%)	
Country-wise Distribution			
Patients	FB (n=959)	RP (n= 759)	Total
Australia	148 (15.4%)	65 (8.6%)	213 (12.4%)
United Kingdom	408 (42.5%)	39 (5.1%)	447 (26%)
United States	403 (42%)	655 (86.3%)	1058 (61.6%)
Sites	FB (n=16)	RP (n=11)	Total
Australia	3 (18.8%)	1 (9.1%)	4 (14.8%)
United Kingdom	7 (43.8%)	1 (9.1%)	8 (29.6%)
United States	6 (37.5%)	9 (81.8%)	15 (55.6%)
Surgeons	FB (n=27)	RP (n=12)	Total
Australia	9 (33.3%)	1 (8.3%)	10 (25.6%)
United Kingdom	13 (48.1%)	1 (8.3%)	14 (35.9%)
United States	5 (18.5%)	10 (83.3%)	15 (38.5%)

Demographics

There was no difference in baseline demographics, including age and gender (Table 1). The mean age of enrolment was 66.1 years in FB TKA (range, 39-80) and 64.3 years in RP TKA (range, 34-80). Gender was evenly distributed across both groups. Female patients comprised 58.8% of FB TKA and 58.2% of RP TKA. 

Mean BMI was found to be 31.9 in FB TKA and 32.3 in RP TKA. The FB TKA group had a slightly higher proportion of patients with a BMI >30 kg/m^2^ (47.9%) compared to the RP TKA group (40.1%). Patients with a BMI >40 kg/m^2^ accounted for 9.9% of FB TKA and 9.1% RP TKA. Other co-morbidities were similar between groups, with no statistically significant differences observed. 

There was no difference in average follow-up between groups. 

Preoperative interventions

Preoperative interventions for both FB TKA and RP TKA are displayed in Table 2. Slightly higher interventions were seen in the FB TKA, but overall, the groups were comparable. 

**Table 2:** Previous treatments for participants with RP TKA and FB TKA FB: fixed bearings; TKA: total knee arthroplasty; RP: rotating platform bearings

Previous Treatment	FB	RP
None	39.80%	33.50%
Intra-articular knee injections	31.90%	32.30%
Total Knee Arthroscopy	31.40%	27.90%
Meniscal	9.50%	10.00%
Total Knee	5.00%	5.00%
Other	4.00%	2.00%
Total Hip Arthroplasty	3.90%	2.90%
Debridement	3.10%	0.00%
Ligament reconstruction	1.60%	5.10%

PROMs

When adjusted for all confounders, no significant difference was found between RP TKA and FB TKA in change from baseline at two years across any parameter in all PROMs investigated (Table 3). With regard to PKIP, no difference was found in either group in any subscale at two years. There were similar improvements from baseline across both groups in all PROMs and their subscales.

**Table 3:** Baseline and two-year PROMs for RP TKA vs FB TKA FB: fixed bearing; RP: rotating platform; CFB: change from baseline; PROMs: patient-reported outcome measures; PKIP: Patient Knee Implant Performance questionnaire [22]; KOOS: Knee Injury and Osteoarthritis Outcome Score [13]; OKS: Oxford Knee Score [12]; EQ-5D-3L: EuroQol five-dimensional questionnaire [20]; KSS: Knee Society Score [21]; TKA: total knee arthroplasty KOOS was available freely for academic research use at the time of our study (prior to 2023); OKS was used under an license agreement with Oxford University Innovation Limited; KSS was used with permission from The Knee Society. EQ-5D-3L was used in accordance with licensing requirements from the EuroQol Research Foundation.

Parameters	FB (N)	RP (N)	Baseline mean					2-year mean					Change from baseline mean					Adjusted change from baseline mean				
			FB	RP	Test	t Statistic	p-value	FB	RP	Test	t Statistic	p-value	FB CFB	RP CFB	Test	t Statistic	p-value	FB Adjusted CFB	RP Adjusted CFB	Test	F Statistic	p-value
PKIP Overall	783	581	27.5	28.4	t-test	1.21	0.227	74.2	75.4	t-test	1.16	0.248	46.7	47.0	t-test	0.27	0.786	46.8	46.9	ANCOVA	0.00	0.983
PKIP Confidence	789	585	3.5	3.8	t-test	2.45	0.014	8.1	8.3	t-test	1.98	0.048	4.6	4.5	t-test	0.34	0.732	4.6	4.5	ANCOVA	0.20	0.655
PKIP Stability	795	594	3.2	3.5	t-test	2.88	0.004	8.3	8.5	t-test	2.38	0.018	5.1	5.0	t-test	0.38	0.703	5	5.1	ANCOVA	0.40	0.528
PKIP Satisfaction	794	596	2.1	2.2	t-test	0.82	0.414	8.1	8.2	t-test	0.53	0.596	6	6.0	t-test	0.08	0.939	6	6	ANCOVA	0.29	0.588
PKIP Modify Activities	796	597	4.1	3.7	t-test	2.80	0.005	6.4	6.6	t-test	1.12	0.264	2.2	2.9	t-test	2.66	0.008	2.4	2.7	ANCOVA	1.30	0.255
KOOS Function in Daily Living	796	598	47.1	50.3	t-test	3.33	0.001	87.1	88.4	t-test	1.61	0.108	40	38.1	t-test	1.74	0.082	39.3	39	ANCOVA	0.05	0.817
KOOS Pain	796	598	42.7	46.3	t-test	4.17	< .0001	87.2	88.1	t-test	1.10	0.273	44.5	41.8	t-test	2.47	0.014	43.4	43.4	ANCOVA	0.00	0.984
KOOS Symptoms	797	599	45.4	48.5	t-test	3.16	0.002	83.4	81.9	t-test	1.73	0.083	37.9	33.4	t-test	3.88	0	36.7	35	ANCOVA	3.19	0.074
KOOS Function in Sport and Recreation	765	554	18	19.1	t-test	0.98	0.327	62.5	67.8	t-test	3.39	0.001	44.4	48.7	t-test	2.51	0.012	46.2	46.3	ANCOVA	0.01	0.924
KOOS Quality of Life	797	599	22.3	23.9	t-test	1.84	0.066	74.8	74.1	t-test	0.54	0.586	52.5	50.1	t-test	1.66	0.097	52.3	50.5	ANCOVA	1.64	0.2
Oxford Knee Total Score (0 - 48)	797	592	21.6	23.7	t-test	5.04	< .0001	41	42	t-test	2.61	0.009	19.4	18.3	t-test	2.37	0.018	18.9	19	ANCOVA	0.05	0.829
EQ5D3L USA Index	797	592	0.6	0.7	t-test	2.48	0.013	0.9	0.9	t-test	3.20	0.001	0.2	0.2	t-test	0.05	0.958	0.2	0.2	ANCOVA	1.68	0.195
EQ5D3L Current Health	796	596	71.3	75.9	t-test	4.95	< .0001	79.6	82.3	t-test	3.13	0.002	8.3	6.4	t-test	1.82	0.069	7.4	7.6	ANCOVA	0.06	0.804
Knee Society Objective Knee Score	494	303	42.5	45.7	t-test	2.21	0.027	90.2	90.9	t-test	0.74	0.457	47.8	45.3	t-test	1.54	0.124	47.4	45.9	ANCOVA	2.51	0.113
Knee Society Satisfaction Score	507	318	11.8	13	t-test	2.57	0.01	34.1	34.4	t-test	0.60	0.552	22.3	21.5	t-test	1.26	0.209	22.1	21.7	ANCOVA	0.44	0.506
Knee Society Functional Activity Score	457	273	36.8	39.7	t-test	2.47	0.014	76.8	79.9	t-test	2.15	0.032	39.9	40.2	t-test	0.16	0.87	40.1	40	ANCOVA	0.00	0.999
Knee Society Expectation Score	510	319	14	14.3	t-test	3.30	0.001	10.7	10.4	t-test	1.49	0.136	-3.3	-4.0	t-test	2.73	0.007	-3.4	-3.8	ANCOVA	2.89	0.09

When adjusted for BMI only, there was a statistical difference favoring RP in several PROMs at two years, including PKIP overall (p=0.003), PKIP confidence (p=0.029), PKIP stability (p<0.013), PKIP modified activities (p<0.0001), OKS total score (p < 0.02), EQ5D3L current health score (<0.001), KSS functional activity (p<0.02), and KSS objective (p=0.021) scores (Table 4). In the same analysis, there was no demonstrated advantage to FB TKA when adjusted for BMI alone at two years. 

**Table 4:** Adjusted changes from baseline with BMI confounder only FB: fixed bearing; RP: rotating platform; CFB: change from baseline; PROMs: patient-reported outcome measures; PKIP: Patient Knee Implant Performance questionnaire [22]; KOOS: Knee Injury and Osteoarthritis Outcome Score [13]; OKS: Oxford Knee Score [12]; EQ-5D-3L: EuroQol five-dimensional questionnaire [20]; KSS: Knee Society Score [21] KOOS was available freely for academic research use at the time of our study (prior to 2023); OKS was used under an license agreement with Oxford University Innovation Limited; KSS was used with permission from The Knee Society. EQ-5D-3L was used in accordance with licensing requirements from the EuroQol Research Foundation.

Parameters	FB (N)	RP (N)	Baseline mean					2-year mean					Change from baseline mean					Adjusted CFB for BMI				
			FB	RP	Test	T Statistic	p-value	FB	RP	Test	T Statistic	p-value	FB CFB	RP CFB	Test	T Statistic	p-value	FB BMI Adjusted CFB	RP BMI Adjusted CFB	Test	F Statistic	p-value
PKIP Overall	783	581	27.5	28.4	t-test	1.21	0.227	74.2	75.4	t-test	1.16	0.248	46.7	47	t-test	0.27	0.786	46.3	47.5	ANCOVA	1.26	0.003
PKIP Confidence	789	585	3.5	3.8	t-test	2.45	0.014	8.1	8.3	t-test	1.98	0.048	4.6	4.5	t-test	0.34	0.732	4.5	4.6	ANCOVA	2.57	0.029
PKIP Stability	795	594	3.2	3.5	t-test	2.88	0.004	8.3	8.5	t-test	2.38	0.018	5.1	5	t-test	0.38	0.703	5	5.2	ANCOVA	3.45	0.013
PKIP Satisfaction	794	596	2.1	2.2	t-test	0.82	0.414	8.1	8.2	t-test	0.53	0.596	6	6	t-test	0.08	0.939	6	6.1	ANCOVA	0.30	0.149
PKIP Modify Activities	796	597	4.1	3.7	t-test	2.80	0.005	6.4	6.6	t-test	1.12	0.264	2.2	2.9	t-test	2.66	0.008	2.4	2.7	ANCOVA	1.81	< .0001
KOOS Function in Daily Living	796	598	47.1	50.3	t-test	3.33	0.001	87.1	88.4	t-test	1.61	0.108	40	38.1	t-test	1.74	0.082	38.9	39.5	ANCOVA	0.64	0.23
KOOS Pain	796	598	42.7	46.3	t-test	4.17	< .0001	87.2	88.1	t-test	1.10	0.273	44.5	41.8	t-test	2.47	0.014	43.2	43.5	ANCOVA	0.13	0.641
KOOS Symptoms	797	599	45.4	48.5	t-test	3.16	0.002	83.4	81.9	t-test	1.73	0.083	37.9	33.4	t-test	3.88	0	36.8	35	ANCOVA	4.95	0.148
KOOS Function in Sport and Recreation	765	554	18	19.1	t-test	0.98	0.327	62.5	67.8	t-test	3.39	0.001	44.4	48.7	t-test	2.51	0.012	44.1	49.2	ANCOVA	10.88	0.077
KOOS Quality of Life	797	599	22.3	23.9	t-test	1.84	0.066	74.8	74.1	t-test	0.54	0.586	52.5	50.1	t-test	1.66	0.097	51.9	50.9	ANCOVA	0.63	0.095
Oxford Knee Total Score (0-48)	797	592	21.6	23.7	t-test	5.04	< .0001	41	42	t-test	2.61	0.009	19.4	18.3	t-test	2.37	0.018	18.7	19.2	ANCOVA	1.95	0.033
EQ5D3L USA Index	797	592	0.6	0.7	t-test	2.48	0.013	0.9	0.9	t-test	3.20	0.001	0.2	0.2	t-test	0.05	0.958	0.2	0.2	ANCOVA	7.38	0.187
EQ5D3L Current Health	796	596	71.3	75.9	t-test	4.95	< .0001	79.6	82.3	t-test	3.13	0.002	8.3	6.4	t-test	1.82	0.069	6.8	8.3	ANCOVA	3.18	< .0001
Knee Society Objective Knee Score	494	303	42.5	45.7	t-test	2.21	0.027	90.2	90.9	t-test	0.74	0.457	47.8	45.3	t-test	1.54	0.124	46.6	47.2	ANCOVA	0.41	0.021
Knee Society Satisfaction Score	507	318	11.8	13	t-test	2.57	0.01	34.1	34.4	t-test	0.60	0.552	22.3	21.5	t-test	1.26	0.209	21.9	22.1	ANCOVA	0.07	0.238
Knee Society Functional Activity Score	457	273	36.8	39.7	t-test	2.47	0.014	76.8	79.9	t-test	2.15	0.032	39.9	40.2	t-test	0.16	0.87	39.3	41.3	ANCOVA	2.14	0.025
Knee Society Expectation Score	510	319	14	14.3	t-test	3.30	0.001	10.7	10.4	t-test	1.49	0.136	-3.3	-4	t-test	2.73	0.007	-3.4	-3.8	ANCOVA	2.29	0.181

The KSS PROM had a smaller sample size by 48.5% in the FB TKA and 58% in the RP TKA. This was due to two separate KSS versions (pre-2011 and post-2011) being used across centres, and analysis was only done on the newer KSS questionnaire. No other PROMs were affected.

Survival rate

Survival rates were comparable between groups at two years. There was a statistically significant difference between groups for revision for any reason; however, the majority of revisions on the RP TKA involved the exchange of the polyethylene insert only. Revisions for any reason at two years in the FB TKA were eight, with a survival rate of 99.1%, and 15 in the RP TKA with a survival rate of 97.9% (p-value: 0.0117) (Table 5). 

**Table 5:** Kaplan-Meier (KM) implant survivorship (removal of any TKA component for any reason) TKA: total knee arthroplasty

	1 Year KM Survivorship (95% CI), N with Later Follow-up (Cumulative Revised)	2 Year KM Survivorship (95% CI), N with Later Follow-up (Cumulative Revised)
FB (N=959)	99.7% (99.0, 99.9), N = 925 (3 Revised)	99.1% (98.3, 99.6), N = 802 (8 Revised)
RP (N=759)	99.1% (98.0, 99.5), N = 720 (7 Revised)	97.9% (96.6, 98.7), N = 558 (15 Revised)

When comparing revisions involving the removal of the femoral and/or tibial component only, there was no statistically significant difference between groups. Revisions involving the removal of the femoral and/or the tibial component at two years in the FB TKA group were four, with a survival rate of 99.6%, and five in the RP TKA group, with a survival rate of 99.3% (p-value: 0.2973) (Table 6). 

**Table 6:** Kaplan-Meier (KM) implant survivorship estimates (removal of the tibial and/or femoral component only)

	1 Year KM Survivorship (95% CI), N with Later Follow-up (Cumulative Revised)	2 Year KM Survivorship (95% CI), N with Later Follow-up (Cumulative Revised)
FB (N=959)	99.9% (99.3, 100.0) N = 927 (1 Revised)	99.6% (98.8, 99.8) N = 806 (4 Revised)
RP (N=759)	99.9% (99.1, 100.0) N = 726 (1 Revised)	99.3% (98.3, 99.7) N = 567 (5 Revised)

Of the 15 revisions in the RP group, 10 involved isolated polyethylene insert exchange without removal of the femoral or tibial components. Indications for isolated polyethylene exchange included infection (n=3), crepitus (n=1), pain (n=3), and stiffness (n=3). In all cases, the polyethylene insert was replaced with the same bearing design, and no conversion between fixed-bearing and rotating platform systems occurred. Only two participants underwent revision for loosening, and both were in the RP TKA group. All revisions and reasons for revisions are presented in Table 7.

**Table 7:** Revision details ^b^ Subjects had a revision involving the removal of the insert prior to having another revision involving the removal of other components. *P.F.C. SIGMA™ Knee System (DePuy Synthes, West Chester, Pennsylvania, United States); **ATTUNE™ Primary Knee System (DePuy Synthes, West Chester, Pennsylvania, United States) FB: fixed bearings; RP: rotating platform bearings; F: femoral; T: tibial; I: insert; P: patella

FB (N=959) 8 Revisions			
Revision Reason	Product	Time (years) at Revision	Components removed^a^
Infection	ATTUNE*	1.7	F, T, I, P
	ATTUNE	0.6	P
Pain	SIGMA**	1.8	I
	SIGMA	1.8	F, T, I, P
	SIGMA	1.7	P
	SIGMA	1	I
		1.5	F, T, I^ b^
Stiffness	ATTUNE	0.4	F, T, I
	ATTUNE	0.9	I
RP (N=759) 15 Revisions			
Revision Reason	Product	Time (years) at Revision	Components removed
Bone Fracture	ATTUNE	1.2	P
	ATTUNE	0.3	F, T, I, P
Crepitus	ATTUNE	0.9	I
Infection	SIGMA	1.1	I
	SIGMA	0.5	I
		1.4	F, T, I, P^ b^
	SIGMA	1.5	F, T, I, P
	ATTUNE	0	I
Loosening	SIGMA	1.7	F, T, I, P
	ATTUNE	1.9	F, T, I
Pain	ATTUNE	1.8	I
	ATTUNE	1.6	I
	SIGMA	0.6	I
Stiffness	ATTUNE	0.4	I
	ATTUNE	0.4	I
	ATTUNE	1.2	I

## Discussion

The purpose of this study was to compare the functional outcomes of rotating platforms and fixed bearing TKA, including use of the novel PKIP PROM, in a post‑hoc pooled analysis of data from two large muti-centre studies. We found that both FB TKA and RP TKA provide excellent improvements in all PROMs over two years. In our initial analysis, adjusting for all confounders, we did not find a statistically significant difference between the rotational platform and fixed bearing prosthesis at baseline and two years across any PROM. When adjusting for BMI, we found that there were statistically significant differences between groups in favour of RP. 

Whilst many PROMs exist for use in TKA studies, PKIP is one of only eight PROMs that have undergone psychometric evaluation [17]. In a recent systematic review, PKIP is the only PROM that has been given an ‘adequate’ rating with regard to evidence in instrument development, whilst others were either ‘low’ or ‘indeterminate’ [23]. Our study is the first to compare RP TKA with FB TKA using PKIP; hence, there are no direct comparisons we can make with other studies. The PKIP allowed measurement of the TKA across several subscales. These included patients’ confidence, satisfaction, stability, and need for medication for activities. We found no difference in overall score or any subscale of PKIP between RP TKA and FB TKA at two years. The only statistically significant differences were seen when adjusting for only BMI.

Our findings concur with similar studies investigating these prostheses using conventional PROMs. Killen et al. found no difference in KSS between RP TKA and FB TKA after 12 years [24]. Theirs is the longest study to date comparing the two prostheses and included 132 patients contributing to 140 surgeries. Unfortunately, given the longer timeframe, there was a significant loss to follow-up (40%), with 20% of patients passing away and an additional 20% withdrawing from the study. The remaining 76 patients completed the KSS PROM. There was no statistically significant difference in the study at the two-year interval between RP TKA and FB TKA. Kalisvaart et al. conducted a randomised control trial (RCT) comparing 76 patients with RP TKA to 152 with FB TKA and found no difference at two or five years in KSS [25]. Bailey et al. conducted an RCT of 352 patients, of whom 163 underwent RP TKA, and 176 underwent FB TKA [26]. The researchers found no difference in OKS postoperatively at two years. Meta-analyses on this topic have found no differences between groups RP TKA and FB TKA. Most recently, a meta-analysis by Chen et al., looking at clinical and functional outcomes between MB TKA and FB TKA at a minimum 10-year follow-up, did not find any differences in KSS [27]. Wang et al. conducted a meta-analysis on 17 studies with a five-year minimum follow-up and did find some improvements in KSS in the MB TKA group; however, they noted significant heterogeneity between studies, including a lack of baseline KSS and small sample sizes [23]. 

We found statistically significant differences in RP TKA and FB TKA in several PROMs at two years when adjusted for BMI. However, these findings should be interpreted cautiously, as this analysis was exploratory and the study was not designed to evaluate BMI as a true effect modifier across predefined BMI subgroups. In our analysis, all differences favoured RP TKA. However, the magnitude of these differences was modest, and it remains uncertain whether they represent clinically meaningful improvements from a patient perspective. Given the increasing prevalence of obesity among patients undergoing TKA, understanding the potential influence of BMI on functional outcomes remains clinically relevant. A higher proportion of patients who are classified as obese (BMI of over 30 kg/m^2^) are undergoing TKA, yet outcomes in this population remain ambiguous [28]. Boyce et al. conducted a systematic review of studies comparing outcomes in morbidly obese and non-obese patients [29]. They found that a higher BMI was associated with a higher complication rate for TKA but reported similar improvements in functional scores. O’Neill et al. conducted an analysis at a single centre on 2,180 patients undergoing primary TKA and found no differences in functional scores between normal weight and overweight groups at six months postoperatively [30]. Their subgroup analysis, however, did suggest worsening functional scores with increasing BMI. Whilst a large study, this was a retrospective study with a limited duration of follow-up. No study to our knowledge has compared outcomes between RP TKA and FB TKA in elevated BMI populations. Our results suggest functional differences between RP and FB TKA may exist at higher BMI; however, we did not perform analysis on sub-groups of different BMI, and this may be the focus of a future project.

Survival rates were excellent in both groups at the two-year follow-up. Revision of either femoral or tibial components remained low in both RP TKA and FB TKA groups at two years. There was a statistically significant difference between FB TKA and RP TKA for the total number of revisions; however, no difference was found when comparing revisions involving the removal of a femoral or tibial implant. Only two patients underwent revision for loosening as the primary indication for revision, and both were in the RP TKA cohort. These findings are similar to other studies investigating survival rates of RP TKA. Meftah et al. conducted a long-term follow-up of over 10 years in 117 patients with RP TKA and found the 10-year survival was 97.7% when revision for any reason was an endpoint [31]. Our study was not able to address the issue of long-term wear, which is the index theoretical advantage of the RP TKA, as the scope of observation was too limited to confidently provide any meaningful conclusions. 

The current study involved a modern implant, the ATTUNE Knee System, which has shown encouraging results for its use in TKA at two years, with no signs of increased revision rates when compared to a well-established implant, the P.F.C. Sigma [32]. This is further supported by findings in the Australian and American National Joint Registries [2,33].

Our study is the largest data set, to our knowledge, comparing RP TKA and FB using prospectively collected data. Our analysis is also the first to compare RP TKA and FB TKA using the new PKIP PROM and adjust for BMI between these two groups. Additionally, the international pool of patients increases the generalisability of our results.

Limitations

There are limitations in our study. In addition to the short timeframe, we did not use any objective tools in our analysis, such as radiographic data, gait analyses, or range of motion. Our KSS PROM dataset was reduced as there was a different version of the KSS PROM used across centres, and analysis was conducted only on the updated KSS questionnaire. This did not affect other PROMs. 

Another limitation of our study is the lack of randomisation in the recruitment process, which may contribute to selection bias and residual confounding. In particular, differences in recruitment across participating centres and surgeons may have contributed to baseline differences between RP TKA and FB TKA cohorts prior to statistical adjustment. Although propensity score adjustment was used to account for measured confounders, unmeasured factors may still have influenced outcomes.

Additionally, the BMI-adjusted analysis should be interpreted cautiously. While differences favouring RP TKA were observed when adjusting for BMI alone, this analysis was exploratory, and the study was not designed to evaluate BMI as a true effect modifier across predefined BMI subgroups. Further investigation in studies specifically designed to examine BMI-related outcomes would be required to confirm these findings.

## Conclusions

The study findings demonstrate similar improvements in the short-term in both RP TKA and FP TKA across all PROMs. Patients reported similar scores in satisfaction, confidence, mobility, and stability in the PKIP PROM for both RP and FB groups. At two years when adjusting for BMI using the 0.05 significance threshold, the following PROMs favoured RP TKA: PKIP Overall, PKIP Confidence, PKIP Stability, PKIP Modified Activities, Oxford Knee Score, Knee Society Objective Knee Score, and Knee Society Functional Activity Score, warranting further investigation.

We did not identify any disadvantage in short-term patient-reported functional outcomes associated with RP TKA at two years. Our findings indicate that, from a patient-reported performance perspective, RP TKA performs comparably to FB TKA in the early postoperative period. Long-term follow-up data from RCTs are needed to establish with certainty any difference between RP and FB designs. 
